# The Natural History of Incidental Colonic Diverticulosis on Screening Colonoscopy

**DOI:** 10.1155/2018/3690202

**Published:** 2018-12-06

**Authors:** Ala I. Sharara, Nathalie Ziade, Rani H. Shayto, Luma Basma O. Rustom, Hamed Chehab, Hussein H. Rimmani, Krystelle Hanna, Jean M. Chalhoub, Fayez S. Sarkis, Mahmoud A. Rahal, Assaad Soweid, Fadi H. Mourad, Kassem Barada, Ali H. Harb

**Affiliations:** Division of Gastroenterology, Department of Internal Medicine, American University of Beirut Medical Center, Beirut, Lebanon

## Abstract

**Background:**

The natural history of colonic diverticulosis is unclear.

**Methods:**

Patients with incidental diverticulosis identified in a previous prospective cross-sectional screening colonoscopy study were evaluated retrospectively for clinic or hospital visit(s) for diverticular disease (DD= acute diverticulitis or diverticular bleeding) using review of electronic health records and patient phone interview.

**Results:**

826 patients were included in the screening colonoscopy study. Three were excluded for prior DD. In all, 224 patients (27.2%; mean age 62.3 ± 8.2) had incidental diverticulosis distributed in the left colon (67.4%), right colon (5.8%), or both (22.8%). Up-to-date information was available on 194 patients. Of those, 144 (74.2%) could be reached for detailed interview and constituted the study population. Over a mean follow-up of 7.0 ± 1.7 years, DD developed in 6 out of 144 patients (4.2%) (4 acute cases of diverticulitis, 1 probable case of diverticular bleeding, and 1 acute case of diverticulitis and diverticular bleeding). Two patients were hospitalized, and none required surgery. The time to event was 5.1 ± 1.6 years and the incidence rate was 5.9 per 1000 patient-years. On multivariate analysis, none of the variables collected at baseline colonoscopy including age, gender, obesity, exercise, fiber intake, alcohol use, constipation, or use of NSAIDs were associated with DD.

**Conclusion:**

The natural history of incidental diverticulosis on screening colonoscopy was highly favorable in this well-defined prospectively identified cohort. The common scenario of incidental diverticulosis at screening colonoscopy makes this information clinically relevant and valuable to physicians and patients alike.

## 1. Introduction

Colonic diverticulosis is the most common incidental lesion on routine colonoscopy [1]. It is estimated that around 15% of individuals will have diverticulosis by the age of 50 and as many as 60% by the age of 80 [1]. Recent evidence indicates that this prevalence is expected to further increase worldwide, possibly due to urbanization and adoption of western lifestyles [2]. The clinical spectrum of symptomatic diverticular disease (DD) ranges from mild abdominal pain to life-threatening complications including perforation or hemorrhage. Complicated DD is a major economic burden accounting for 312,000 hospital admissions, 1.5 million-days of hospital stay, and more than 2.6 billion dollars in 2004 [3, 4]. The true economic burden may however be higher given that uncomplicated disease is often diagnosed and managed in ambulatory settings [5].

It has been suggested that most patients with diverticulosis remain asymptomatic and that 10-25% eventually develop diverticulitis [6, 7]. However, despite the poor quality of evidence, these numbers continue to appear in surgical and medical guidelines [6, 8]. A recent retrospective study from the Veterans Administration has challenged this view suggesting an actual incidence of acute diverticulitis as low as 4% [9]. The study had some limitations including selection of patients, a restrictive definition of outcome and lack of assessment of diverticular bleeding, an important complication which is estimated to occur in 3-5% of patients with diverticulosis [10].

The aim of this retrospective cohort study is to determine the incidence of complicated DD in patients with incidental diverticulosis identified in a previous prospective cross-sectional screening colonoscopy study in average-risk patients, originally designed to investigate the prevalence and risk factors of colorectal neoplasia as well as diverticulosis [11, 12].

## 2. Methods

This was a retrospective cohort study conducted to determine the incidence of DD (defined as acute diverticulitis, diverticular hemorrhage, or secondary stricturing or fistulizing disease necessitating surgery) in patients with incidental diverticulosis identified in a previous prospective screening colonoscopy cross-sectional study. The latter was conducted between 2005 and 2010 in average-risk individuals older than 50. It prospectively examined the prevalence and risk factors for colonic neoplasia [11] as well as the prevalence, distribution, and risk factors of diverticulosis (as part of its original design) in an asymptomatic population [12].

A retrospective review of electronic health records of all patients with previously documented diverticulosis was conducted looking specifically for clinic or hospital visits and/or cross-sectional abdominal imaging over the follow-up period from index colonoscopy to phone interview. In addition, patients were contacted individually and asked about the occurrence of abdominal pain necessitating clinic and/or hospital visit and where a diagnosis of “diverticula-related problem” was suggested and antibiotics prescribed, or the occurrence of significant hematochezia requiring emergency room visit or hospital admission. To minimize recall bias, immediate family members were asked the same question when available. The study was approved by the Institutional Review Board.

## 3. Results

A total of 826 consecutive patients were included in the original prospective screening study. Three patients were excluded because of prior history of DD. Incidental diverticulosis was noted in 224 of 823 patients (27.2%) (mean age 62.3 ± 8.2 years; M: F=1.15). Diverticula were isolated to the left colon in 151 patients (67.4%), were right-sided in 13 (5.8%), and diffuse (right and left) in 51 (22.8%). Nine patients (4%) had missing data about colonic location of the diverticula. Of the 224 patients, 194 patients had up-to-date medical records and constituted the study population. Of those, 144 (74.2%) could be reached by telephone and consented to provide additional information. Nine patients were deceased at the time of follow-up due to unrelated causes.

**Figure 1:**
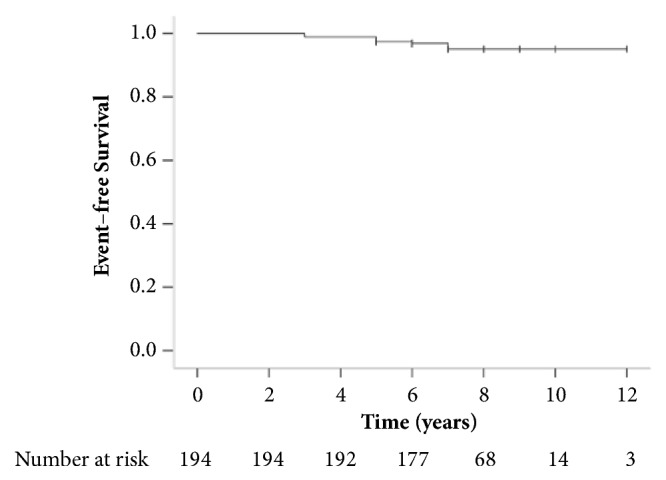
Kaplan-Meier curve of the cumulative incidence of diverticular disease.

Overall, DD developed in 6 patients (4.2%): 4 patients developed acute diverticulitis (2.8%), 1 (0.7%) had possible diverticular bleeding and one patient (0.7%) had both diverticular bleeding and acute diverticulitis. Acute diverticulitis was confirmed by computerized tomography in 4 out of the 5 patients that were contacted. One patient was admitted to another hospital and received antibiotics for a putative diagnosis of acute diverticulitis without cross-sectional imaging (information provided by the patient and family at phone interview). None of the patients with acute diverticulitis required surgery or percutaneous intervention. The single patient with probable diverticular bleeding presented with one-day history of recurrent moderate hematochezia. His previous colonoscopy was negative except for left-sided diverticulosis. He was hospitalized for 48 hours, remained stable and did not require angiography or purge colonoscopy. Overall, 1 of the 6 patients with DD (16.7%) was hospitalized and the rest were managed conservatively. The records of patients who could not be reached by phone were reviewed and showed acute diverticulitis in two patients. One was documented by computerized tomography while the other was managed conservatively on outside basis. The mean follow-up period was 7.0 ± 1.7 years (median 7 years). The incidence of DD was 5.9 per 1000 patient-years and the time to event was 5.0 ± 1.6 years (median 5 years) (Figure 1). On univariate and multivariate analysis, none of the variables collected at baseline colonoscopy including age, gender, obesity, exercise, alcohol, constipation, or the use of NSAIDs or fibers were associated with DD.

## 4. Discussion

Colonic diverticulosis is one of the most common conditions affecting the colon. In 1968, a postmortem study from Belfast of 300 unselected colons identified diverticula in 37% of specimens [13]. Prevalence increased with age of death reaching nearly 25% of colons by age 50 to as many as 40-50% by ages 70-80. Barium enema may be the best premortem method to identify the true prevalence of colonic diverticulosis but is understandably not routinely performed in asymptomatic individuals. The wide adoption of colonoscopy as an effective tool in screening for colorectal neoplasia offers a unique opportunity to study the prevalence, risk factors, and natural history of colonic diverticulosis. A recent prospective screening colonoscopy study from the USA involving more than 600 patients reported a prevalence of diverticulosis of 42% with predominance in the sigmoid colon [14]. Risk factors included age, male gender, and a higher body mass index (BMI). Overall, prevalence increased with age (40% in the 5^th^ decade, and 58% in older patients). A higher percentage of proximal colon diverticulosis was noted in black persons. The prevalence of asymptomatic diverticulosis in our study population was 27.1% (age-adjusted prevalence was 21.4%, 41.8%, and 45.8% in the 5^th^, 6^th^, and 7^th^ decade, respectively [11]) mirroring that of a recent Dutch colonoscopy study [15]. Diverticulosis was more common in the recent prospective US screening study (42%) [14] but this difference may be attributable to a difference in age distribution, higher BMI, and probably other yet unidentified risk factors [16, 17].

The true natural history of colonic diverticulosis is largely unclear [18]. The lifetime risk of acute diverticulitis has been estimated at 10%-25% but these figures were based on older literature without precise studies on prevalence of the uncomplicated diverticula in asymptomatic populations. For example, in the large Health Professionals Follow-up Study, Strate et al. examined the incidence and risk factors for DD [16, 19–21]. These studies were however all limited by the lack of preassessment of the prevalence of asymptomatic diverticulosis in the study population (unknown true denominator) relying primarily on the participants' self-diagnosis of diverticulosis or its complications for exclusion. Given the largely asymptomatic nature of the disease, such studies do not to provide accurate evidence about the true natural history of diverticulosis.

Recently, a large retrospective study from the Veterans Administration examined 2222 patients with baseline diverticulosis at colonoscopy [9]. Over an 11-year period, 23 patients (1%) developed rigorously-confirmed diverticulitis for an incidence rate of 1.5 per 1000 patient-years. The incidence rate increased to 6.0 per 1000 patient-years when cases of diverticulitis without confirmatory imaging or surgery were included. The median time-to-event was 7.1 years. The study was retrospective and consisted predominantly of male patients (97%). Given that most cases of diverticulitis are uncomplicated in nature and are commonly managed in an ambulatory setting [5], often without imaging, it is possible that the authors underestimated the true incidence of acute diverticulitis. In addition, the study population was retrospectively identified based on colonoscopic reports and consequently this may underrepresent the population-at-risk as endoscopists may not systematically record this common incidental finding at colonoscopy. Unlike our study, diverticulosis was identified at colonoscopy* for any indication* and was not restricted to an average-risk screening population without history of DD (screening indication in less than half the cases). Lastly, there was no information on the incidence of diverticular bleeding or hemorrhage in the study population.

Niikura et al. examined the bleeding risk in 1514 patients with asymptomatic diverticulosis over a 12-year period [10]. The median follow-up was 3.8 years. Diverticular bleeding occurred in 35 patients (definitive in 7 and presumptive in another 28). The cumulative incidence of diverticular bleeding was 0.21% at 12 months, 2.2% at 60 months and 9.5% at 120 months and the median time-to-event interval was 4.2 years. The overall incidence rate of bleeding was 0.46 per 1000 patient-years. On multivariate analysis, bilateral diverticulosis and age ≥70 were significant risk factors for bleeding. The limitations of the study were the retrospective design and the all-indication colonoscopy study population. In addition, there was no information on the incidence of acute diverticulitis in that study.

The strengths of our study include the well-defined prospectively-identified population, the long-duration of follow-up of the study cohort, and ascertainment of all relevant endpoints by review of medical records as well as direct patient and family interview, potentially offering the opportunity to uncover uncomplicated cases of acute diverticulitis, diagnosed and managed in a different healthcare setting or in ambulatory care without cross-sectional imaging or hospital admission. Our study has few limitations. Despite ongoing care and accessible medical records at our hospital, it is conceivable that some patients who could not be contacted by telephone had DD diagnosed and managed at another healthcare facility. The incidence rate is however unchanged when these patients are excluded (data not shown). Other limitations include the retrospective nature of the study and the relatively small size of the total study cohort. The high concordance with the above-cited two natural history studies [9, 10] supports our study results

## 5. Conclusion

Using a well-defined prospectively identified cohort, we show that the natural history of incidental diverticulosis in average-risk individuals 50 years or older undergoing colonoscopy screening is highly favorable with an incidence rate of less than 6 per 1000 patient-years. The common scenario of incidental diverticulosis at screening colonoscopy makes this information clinically relevant and valuable to physicians and patients alike.

## Data Availability

The data used to support the findings of this study are available from the corresponding author upon request.
